# Effect of Finger Gnosis on Young Chinese Children’s Addition Skills

**DOI:** 10.3389/fpsyg.2020.544543

**Published:** 2020-09-30

**Authors:** Li Zhang, Wei Wang, Xiao Zhang

**Affiliations:** ^1^School of Sociology and Psychology, Central University of Finance and Economics, Beijing, China; ^2^Faculty of Education, The University of Hong Kong, Hong Kong, China

**Keywords:** finger gnosis, addition skills, number line estimation, young children, the addition strategy

## Abstract

Evidence has revealed an association between finger gnosis and arithmetic skills in young Western children, however, it is unknown whether such an association can be generalized to Chinese children and what mechanism may underlie this relationship. This study examines whether finger gnosis is associated with addition skills in young Chinese children and, if so, what numerical skills could explain this correlation. A total of 102 Chinese children aged 5–6 years were asked to complete finger gnosis and addition tasks in Study 1. Results showed that finger gnosis was significantly associated with addition performance. However, no significant correlation was found between finger gnosis and the use of finger counting in solving addition problems. Moreover, girls’ finger gnosis was better than boys’, and children with musical training demonstrated better finger gnosis than those without. In Study 2, 16 children with high finger gnosis and 20 children with low finger gnosis were selected from the children in Study 1 and asked to perform enumeration, order judgment, number sense, and number line estimation. Children with high finger gnosis performed better in number line estimation than their counterparts with low finger gnosis. Moreover, the number line estimation fully mediated the relationship between finger gnosis and addition performance. Together, these studies provide evidence of a correlation between finger gnosis and addition skills. They also highlight the importance of number line estimation in bridging this association.

## Introduction

Finger use for math calculations is natural and intuitive (Jordan et al., 2008). A large body of research has found that fingers (e.g., finger gnosis, finger tapping, and finger counting) play an important role in arithmetic processing (e.g., Noël, 2005; Gracia-Bafalluy and Noël, 2008; Costa et al., 2011; Lafay et al., 2013; Crollen and Noël, 2015b; Soylu and Newman, 2016). Finger gnosis, also termed “finger sense” or “finger schema” (Penner-Wilger and Anderson, 2008), is defined as the ability to identify fingers without visual involvement. Emerging evidence has suggested an association between finger gnosis and arithmetic skills in young Western children, however, it is not clear whether such an association can be generalized to Chinese children and what mechanisms may underlie this relationship. In this study, we examine the correlation between finger gnosis and addition skills in young Chinese children and the mechanism underlying this relationship.

Findings supporting the relationship between finger gnosis and arithmetic skills have originated from cross-sectional, longitudinal, training, and neuropsychological studies in young Western children (e.g., Rusconi et al., 2005; Costa et al., 2011; Reeve and Humberstone, 2011; Crollen and Noël, 2015b). For instance, Fayol et al. (1998) found that scores on a neuropsychological battery of somatosensory integrity of the sensory cortex, which included a finger gnosis test, represented a longitudinal predictor of arithmetic performance in 5–6-years-old children in France. Based on a longitudinal sample of first graders in Belgium, Noël (2005) reported that finger gnosis predicted numerical performance (including later addition, subitizing, number writing and digit comparison, collection comparison, and finger counting) 1 year later. Penner-Wilger et al. (2007) discovered that finger gnosis directly predicted number system knowledge and indirectly predicted calculation skills in Canadian first graders; they speculated that children with high finger gnosis solve mathematics problems by using their fingers as representational tools. Reeve and Humberstone (2011) explored the relationship between non-motoric finger gnosis, which does not involve motor movement (e.g., pointing), and single-digit addition operations in 5–7-years-old Australasian children. Their findings provided direct evidence for the importance of measuring non-motoric finger gnosis when predicting arithmetic ability. An electrostimulation study of Gerstmann syndrome (Roux et al., 2003) found that electrostimulation in the angular gyrus, supramarginal gyrus, or close to the intraparietal sulcus produced disturbances in finger recognition and calculation abilities. This finding suggests that finger gnosis and arithmetic calculation may share common neural mechanisms. Similarly, a functional magnetic resonance imaging study by Andres et al. (2012) revealed that finger discrimination and mental arithmetic induced a similar pattern of parietal activity in adults.

Recently, several researchers (Poltz et al., 2015; Long et al., 2016; Wasner et al., 2016) have reported that the magnitude of the correlation between finger gnosis and arithmetic skills might be smaller than previously assumed. For example, Poltz et al. (2015) found that the correlation of finger gnosis with numerical abilities (numerals knowledge, counting skills, and calculation) was weaker than its correlation with non-verbal intellectual ability in German preschool children. Similarly, Wasner et al. (2016) recruited a sample of German first graders (mean age: 6.47 years) and found that finger gnosis predicted a unique and relevant but only a small proportion (1–2%) of the variance in arithmetic performance beyond a pool of general cognitive abilities and numerical precursor competencies.

Moreover, in a study with Australasian first and second graders, Long et al. (2016) found no meaningful association between finger gnosis and either counting or arithmetic skills after controlling for the effects of age, however, participants were from primary schools. For younger children who have not entered primary school, finger gnosis may play a more critical role because it helps them to construct the counting system and acquire number concepts. By contrast, the importance of finger gnosis could decline after children enter primary school because finger use is often regarded as an inefficient strategy at this level. For instance, a longitudinal study by Jordan et al. (2008) examined changes in the frequency of finger use in learning number combinations from the beginning of kindergarten (mean age = 5.7 years) to the end of second grade. Finger use was found to be most adaptive when children were first learning number combinations in kindergarten, but this benefit lessened over time. Indeed, in the study by Long et al. (2016), finger gnosis correlated moderately with the arithmetic ability (*r* = 0.43). However, once age was controlled, the relationship between finger gnosis and calculation ability became negligible, accounting for just 1.4% of the variance, suggesting the importance of age in the correlation between finger gnosis and arithmetic ability. Hence, we can speculate that the disassociation between finger gnosis and addition skills in Long et al. (2016) may be due to children’s less frequent use of a finger strategy in solving arithmetic problems after entering primary school.

Contrary to our speculation, Newman (2016) studied a sample of US children and found that the association between finger sense and addition skills did not exist in the younger group (5–8-years-old children) but in the older group (9–12-years-old children). However, Newman’s study had a number of critical limitations, such as small sample size (*N* = 34) and a timed addition test that was extraordinarily difficult for 5–8-years-old children (i.e., the accuracy rate was approximately 50% on average for this age group). Hence, Newman’s finding remains to be verified with a larger sample and by adopting more appropriate tasks.

Overall, most studies have suggested a relationship between finger gnosis and arithmetic skills in young Western children, although a few studies have indicated that this correlation may not be strong. Scholars have also expressed interest in the mechanisms underlying the association between finger gnosis and arithmetic skills. Three explanations have dominated the field to this point.

First, *the functionalist explanation* asserts that the correlation between finger representation and mathematical ability is due to children’s experience and development. The link between finger gnosis and math ability formed experientially throughout normal development to represent quantities and perform counting and arithmetic procedures (Butterworth, 1999). Gracia-Bafalluy and Noël (2008) argue that their study can provide support for the functional link between finger gnosis and number skills in a training study. After the finger training, which consisted of 2 weekly sessions of 30 min each for 8 weeks, children with poor finger gnosis performed significantly better than those in the control group on finger gnosis, representation of numerosities with fingers, and quantification tasks. These results indicate that improving finger gnosis can provide useful support for learning mathematics.

Second, *the localizationist explanation* posits that the association between finger gnosis and mathematical ability is caused by adjacent brain areas in the parietal lobe that are responsible for the two skills (Dehaene et al., 2003). Simon et al. (2002) found that regions in the human parietal cortex activated for calculation are adjacent to those for grasping and pointing.

Third, *the redeployment explanation* suggests that finger gnosis is associated with mathematical ability because of an overlap between the functional representations of fingers and mathematics (Penner-Wilger and Anderson, 2008). Specifically, one of the functional circuits originally evolved for finger representation is redeployed to support number representation and finally serves both functions. By comparing functional neuroimaging data across cognitive domains, Penner-Wilger and Anderson (2011) identified a region within the left precentral gyrus contributing to finger gnosis and number representation. With a variety of number and finger tasks, functional imaging studies have consistently shown overlapping activation in parietal regions (Andres et al., 2007, 2012). In a series of experiments, Rusconi et al. (2005) found that rTMS over the left angular gyrus disrupted magnitude comparison and finger gnosis in adults, implying that a common neural substrate exists between number and fingers. Using direct cortical stimulation, Roux et al. (2003) identified a site in the left angular gyrus that produced acalculia and finger gnosis.

A careful inspection of the three explanations suggests that they are not mutually exclusive. Specifically, *the redeployment explanation* is actually an integration of functionalism and localism. Close or overlapping neural foundations of finger gnosis and arithmetic skills are the common emphases of the redeployment and localizationist views. Dynamic cognitive use shaped by experience and development is the common emphasis of the redeployment and functionalist views. In this sense, Penner-Wilger and Anderson (2013) suggest that it is difficult to distinguish between redeployment and functionalism. For example, two dual-task studies have revealed that finger movements interfere with addition (Michaux et al., 2013; Soylu and Newman, 2016), which can provide support for both the redeployment and functionalism views.

The purpose of the present study is twofold. First, we seek to examine whether an association between finger gnosis and addition skills exists in young Chinese children. So far, it is unknown whether the correlation identified between finger gnosis and addition skills in Western children can be generalized to Chinese children. Chinese children tend to use a culturally unique one-hand-finger-counting strategy. They often count 1–5 on the right hand in a way that is familiar to their peers in North America and most European countries. However, they usually count 6–10 using symbolic sign gestures continued on the same hand (Domahs et al., 2010; Morrissey et al., 2016). Finger counting is inherently time-consuming, so using symbolic sign gestures to represent 6–10 may be beneficial for children to acquire a flexible representation of fingers. As Reeve and Humberstone (2011) proposed, finger gnosis may develop through two stages: (1) acquisition of a flexible representation of fingers and (2) a flexible ability to use fingers as a cognitive tool in number cognition. Young Chinese children’s flexible representation of fingers may exert a positive role in their addition skills before they enter primary school. Therefore, we hypothesized that there was a significant association between finger gnosis and addition skills. Studying such an association may provide further evidence for the importance of finger gnosis in children’s arithmetic development in a culture different from the West.

To accomplish the first objective, the present research offers one improvement over prior work. We explore the correlation between children’s finger gnosis and their use of a finger-counting strategy in solving addition problems. Finger counting plays an important role in early mathematical calculation skill development (Moeller et al., 2012). It differs from other strategies such as memory retrieval, verbal counting, and decomposition (e.g., Siegler, 1999) in that it provides preliminary and grounding sensorimotor experiences for children’s perceptions of quantities. Moreover, finger counting is conducive to representing and executing quantities, which accelerates the transition between early non-verbal representations and traditional symbolic representations. Studies have shown that finger counting could bridge an accurate correlation between number combination and its solution (e.g., Siegler and Shipley, 1995; Jordan et al., 2008). Scholars have also found that the use frequency of a finger-counting strategy in preschool and first-grade children is positively correlated with addition performance (e.g., Jordan et al., 1994, 2008; Roesch and Moeller, 2015). Based on previous studies (e.g., Penner-Wilger et al., 2007), we hypothesize that finger gnosis is correlated with finger counting.

The second purpose is to explore whether basic number processing mediates the relationship between finger gnosis and addition skills. Most studies have examined the direct link between finger gnosis and arithmetic skills; to the best of our knowledge, only one study (Penner-Wilger et al., 2007) has explored the indirect link between finger gnosis and arithmetic skills. The study revealed that finger gnosis had an indirect effect on arithmetic skills via the mediating role of children’s number system knowledge, which included counting, ordering, recognizing numerals, sequencing, and place value (Penner-Wilger et al., 2007). However, it is unclear whether other number processing abilities could mediate the link between finger gnosis and arithmetic skills. Previous studies have shown that children’s mathematical achievements are closely associated with their number processing abilities, including enumeration (e.g., Hannula-Sormunen et al., 2015), numerical ordering (e.g., Lyons and Beilock, 2011), number sense (e.g., Halberda et al., 2008; Mazzocco et al., 2011; Starr et al., 2017), and number line estimation (e.g., Siegler and Booth, 2004; Muldoon et al., 2013; Bos et al., 2015). In the present research, we explore whether the association between finger gnosis and addition skills is mediated by number processing abilities, including enumeration, number ordering, number sense, and number line estimation. Compared with Penner-Wilger et al. (2007), we expanded the number system knowledge by including number sense and number line estimation. Although it is theoretically important to examine the differential roles of finger gnosis in multiple domains of arithmetic operations (addition, subtraction, multiplication, and division) that involve very different strategies (Zhou et al., 2011), the present research focused solely on addition skills.

To address the discussed objectives, we examined whether finger gnosis was associated with young Chinese children’s addition skills and the use of a finger-counting strategy in solving addition problems in Study 1. We tested whether the relation between finger gnosis and addition skills could persist after controlling for the child’s sex and experience of playing musical instruments. Previous research has shown that men performed more quickly and regularly than women in finger tapping (e.g., Nicholson and Kimura, 1996; Schmidt et al., 2000; Prigatano et al., 2008). There is also evidence showing that children who played musical instruments (e.g., piano or guitar) performed better on finger gnosis tests and numerical tasks than children who did not (Gracia-Bafalluy and Noël, 2008). We, therefore, included the child’s sex and musical training experience as control variables in the present research. In Study 2, we further examined whether children’s numerical abilities mediated the relationship between finger gnosis and addition skills.

## Study 1

### Materials and Methods

#### Participants

Participants were 111 children recruited from the affiliated kindergarten of a university in Southwest China. Nine children were excluded because they did not complete all tests; thus, 102 children (51 boys and 51 girls) were included in the analysis. Their ages ranged from 60 to 83 months (*M* = 67.68, *SD* = 4.59 months). Among these children, 45 children reported that they were playing musical instruments such as piano, guitar, and flute. Children received stickers after each round of testing. Parents were asked to give their written consent to their child’s participation in advance. The study procedure was approved by the Institutional Review Board of Southwest University and complied with the ethical guidelines of the American Psychological Association.

#### Procedures and Measures

Children completed the finger gnosis task first and then the addition task on an individual basis in a sound-attenuated room.

##### Finger gnosis

The finger gnosis task was adapted from Gracia-Bafalluy and Noël (2008) and Reeve and Humberstone (2011). Each child sat facing the experimenter and placed his/her hand palm-down in a special box on a table with fingers spread. The box was open on the experimenter’s side with a 10 × 4 cm hole on the child’s side. The hole was large enough for the child to put his/her hand through but small enough for the child not to be able to see his/her hand. In each trial, the experimenter gently touched the child’s fingernail(s) with a fingertip and then removed the box and asked the child to identify which finger or fingers had been touched. The test consisted of two parts. The first part was administered on each child’s dominant hand (i.e., the hand the child used to write), and the second part was on the non-dominant hand. Each part consists of three blocks. In the first block, the experimenter touched only one finger, and each finger was touched twice (i.e., 5 × 2 = 10 trials). In the second block, the experimenter touched two fingers simultaneously, and each finger was touched twice (i.e., five trials). In the third block, the experimenter touched two fingers successively, and each finger was touched twice (i.e., five trials). Therefore, 40 trials were presented in total (i.e., 20 trials each for the dominant and non-dominant hand). Finally, the number of correct trials was computed as the performance in the finger gnosis task.

##### Addition task

The addition task contained 30 addition problems in which the addends varied from 2 to 7. Twenty-two problems had sums up to 10 (e.g., 3 + 7), and the remaining eight problems had sums ranging from 11 to 13. No problems had identical addends (e.g., 2 + 7 and 7 + 2). Each addition problem was presented visually in a card. Each child accepted a given order of the problems and was presented with one problem at a time. Children were allowed to use his/her fingers or count aloud to solve each problem. No time limit was instituted on the problems.

Children’s addition performance was indexed by accuracy (i.e., the percentage of problems solved correctly). To measure strategy use, the experimenter observed the children closely and recorded any overt signs of strategy use (e.g., counting aloud, silently moving lips, or using fingers) in solving each of the 30 problems. In the absence of overt behaviors, the experimenter asked the child how he or she had “figured [the problem] out.” Overt behavior and verbal explanations were each used to determine the strategy a child used to solve each problem. Based on previous studies (e.g., Rittle-johnson and Siegler, 1999; Laski et al., 2013), five strategies were coded: finger counting, oral counting, retrieval, decomposition, and other. A strategy was categorized as retrieval if the child reported that he or she “just knew” the answer, and the response speed was relatively fast compared with other strategies. A strategy was categorized as “other” when the child said “I don’t know” or reported having guessed the answer. The retrieval strategy involves recalling the solution to an arithmetic problem from memory. Decomposition involves decomposing a problem into simpler problems; for example, to solve 5 + 7, a child might first add 5 + 5 to get 10 and then add two to arrive at 12. Four experimenters, who did not know our research hypotheses, coded children’s strategies. Before coding, they were trained about how to assign one of five possible codes to addition problems. As long as they were not sure about how to code one problem, the researchers and four coders discussed carefully together and then gave a final code.

#### Statistical Analysis

We first conducted a series of 2 (sex: boy vs. girl) × 2 (musical training: yes vs. no) analysis of variance (ANOVA) tests to examine sex and musical training differences in finger gnosis and addition skills. In these ANOVAs, sex and musical training were the between-subjects variables with age as a covariant; finger gnosis, addition accuracy, and frequency of each strategy were the dependent variables. We then carried out zero-order correlations and multiple regressions to evaluate whether finger gnosis was associated with children’s addition performance and strategy use. All data analyses were conducted in SPSS 21.0.

### Results

Results of the 2 × 2 ANOVAs with age as a covariant showed that the main effect of sex on finger gnosis was significant, *F*(1, 102) = 9.23, *p* = 0.003, η^2^ = 0.087, Cohen’s *d* = 0.53, with lower finger gnosis in boys than in girls. The main effect of musical training was also significant, *F*(1, 102) = 4.72, *p* = 0.032, η^2^ = 0.046, Cohen’s *d* = 0.47. Children who had musical training performed better on finger gnosis than those with no musical training. No significant interaction effect was observed between sex and musical training (*p* = 0.447).

In addition, a significant sex main effect was observed in the use of retrieval strategies, *F*(1, 102) = 6.07, *p* = 0.016, η^2^ = 0.059, Cohen’s *d* = 0.54. Boys tended to use retrieval strategies more frequently than girls. No significant main effect of musical training or interaction effect was observed between sex and musical training (*p*s > 0.928). Analyses of addition accuracy and the other three strategies showed no significant main effects of sex or musical training or interaction effects between sex and musical training (*p*s > 0.111). Descriptive statistics are listed in Table 1.

**TABLE 1:** Descriptive statistics in Study 1.

	Sex				Experience of playing musical instruments				Total	
			
	Boy		Girl		Yes		No			
				
	*M*	*SD*	*M*	*SD*	*M*	*SD*	*M*	*SD*	*M*	*SD*
Finger gnosis	75.01	11.55	80.86	10.94	80.89	12.03	75.61	10.65	77.94	11.53
Addition accuracy	76.88	26.30	76.29	22.69	80.00	24.80	73.89	24.03	76.59	24.44
Finger counting	36.86	36.08	49.61	37.86	44.03	36.06	42.22	39.31	43.23	37.35
Oral counting	16.41	24.67	25.56	34.35	22.92	30.98	18.52	29.13	20.98	30.11
Retrieval	25.82	30.39	11.96	20.34	17.60	23.70	20.52	30.18	18.89	26.66
Decomposition	4.18	9.63	1.83	4.28	2.16	4.52	4.07	10.07	3.01	7.51

**Legend for finger gnosis and addition accuracy is the percentage of correct trials (%); legend for each strategy is the percentage of using the strategy (%).**

We also analyzed correlations between finger gnosis and addition skills and strategies. We only considered correlations that remained significant after applying Bonferroni–Holm corrections for multiple tests (resulting in a reduced alpha = 0.05/21 or 0.0024). Correlations are presented in Table 2. Addition accuracy was significantly correlated with finger gnosis as well as the child’s age and use of retrieval and decomposition strategies. However, the use of a finger-counting strategy did not correlate with finger gnosis but correlated negatively with the use of oral counting and retrieval strategies.

**TABLE 2:** Correlations among the variables in Study 1.

	1	2	3	4	5	6	7
1. Finger gnosis	−						
2. Addition accuracy	0.346**	−					
3. Finger counting	0.063	–0.042	−				
4. Oral counting	0.167	0.161	−0.513***	−			
5. Retrieval	–0.010	0.395**	−0.533***	–0.066	−		
6. Decomposition	0.030	0.254**	−0.286**	–0.062	0.327**	−	
7. Child age	0.277**	0.341***	–0.032	–0.076	0.217*	**0**.300**	−

Correlations that are significant after Bonferroni–Holm correction for multiple testing (alpha = 0.05/21 is 0.0024) are indicated in boldface. *p < 0.05. **p < 0.01. ***p < 0.001.

Finally, we conducted multiple regressions to examine whether finger gnosis was associated with addition accuracy after controlling for the child’s age and the use of a retrieval strategy. Results are shown in Table 3. Finger gnosis significantly predicted addition accuracy even after controlling for covariates. The proportion of variance in addition accuracy explained by finger gnosis was 7.7%.

**TABLE 3:** Regression model predicting addition accuracy.

	Addition accuracy				
	
	*B*	*SE*	β	*t*	*P*
Use frequency of retrieval strategy	0.329	0.079	0.359	4.15	0.000
Finger gnosis	0.635	0.186	0.300	3.41	0.001
Age	0.952	0.475	0.180	2.00	0.048

### Discussion

Consistent with our hypothesis, a positive correlation was found between finger gnosis and addition skills in 5–6-years-old Chinese children. Furthermore, finger gnosis explained a unique and substantial proportion of variance in addition performance after controlling each child’s age, sex, experience of musical training, and strategy use. These findings provide evidence for the close association between finger gnosis and young Chinese children’s addition performance. Unexpectedly, children’s use of a finger-counting strategy in solving addition problems was not associated with finger gnosis and children’s addition performance. We went on to examine the possible mechanism underlying the close association between finger gnosis and addition performance in Study 2, such as number processing abilities.

This study also observed sex differences in finger gnosis; girls demonstrated better finger gnosis than boys. Experience playing musical instruments was also found to be related to finger gnosis, with children who had more musical training demonstrating better finger gnosis. The use of retrieval strategies also revealed a sex difference, indicating that boys were more likely to use retrieval strategies than girls. We discuss these findings further in section “General Discussion.”

## Study 2

Study 2 aimed to examine the roles that basic number processing abilities play in explaining the correlation between finger gnosis and children’s addition skills. To this end, we conducted four basic number processing tests, namely the enumeration task, the number sense task, the order judgment task, and the number line estimation task.

### Materials and Methods

#### Participants

Based on children’s finger gnosis scores in Study 1, two groups of children were selected for participation in Study 2. One group had high finger gnosis and included 7 boys and 13 girls (top 20%; accuracy ranging from 0.87 to 0.97). Their ages ranged from 61 to 78 months (*M* = 70.30, *SD* = 4.37 months). The other group had low finger gnosis and included 10 boys and 6 girls (bottom 20%; accuracy ranging from 0.42 to 0.67). Their ages ranged from 61 to 75 months (*M* = 66.63, *SD* = 4.80 months). For the low finger gnosis group, 20 children were selected initially, but 4 did not complete all tasks; thus, 16 children were analyzed. The ratio of boys to girls in the two groups did not differ significantly, χ^2^(1) = 2.697, *p* = 0.101. An independent sample *t*-test revealed a significant difference in finger gnosis between the two groups, with higher accuracy for the high finger gnosis group (*M* = 0.91, *SD* = 0.03) than for the low finger gnosis group (*M* = 0.62, *SD* = 0.07), *t*(35) = −15.808, *p* < 0.001.

#### Procedure and Materials

Four computerized tasks were administered to the children in Study 2. Three tasks (enumeration, number sense, and number line estimation) were administered online^1^. The children completed all tasks individually in a sound-attenuated room while facing a computer screen from a distance of approximately 60 cm. The experiment included two sessions: in the first, children finished the enumeration and order judgment tasks in random order; in the second, they completed the number sense and number line estimation tasks in random order. The entire experiment was compiled using E-prime.

##### Enumeration task

The stimuli were displayed on a computer screen with black dots (1 cm in diameter) distributed randomly in the central screen box (10 × 10 cm). The number of black dots in the box varied from 1 to 6. The dots were repeated five times, resulting in 30 trials. Each trial was presented randomly. In each trial, the black dots were displayed for 300 ms after a fixation point “+” was presented for 500 ms. Children were instructed to orally state the number of black dots quickly and accurately, and the experimenter helped each child press the corresponding number response. Each child completed six practice trials before the formal experiment. The proportion of problems solved correctly indexed children’s performance.

##### Order judgment task

This task was adapted from Turconi et al. (2006). The display shown in each trial consisted of a pair of single-digit Arabic numbers ranging from 1 to 9, one on the left and one on the right of the screen. Eight quantity combinations were presented, including those with far distance (2–5, 3–6, 4–7, and 5–8) and those with close distance (2–3, 3–4, 6–7, and 7–8). All pairs were presented in ascending (e.g., 2 3) and descending order (e.g., 3 2), resulting in 16 pairs. Each pair was repeated four times, resulting in a total of 64 trials, and divided into two blocks. In each trial, the fixation “+” was first presented for 500 ms followed by two numbers. The numbers remained on the screen until a button was pressed. The intertrial interval was 500 ms. Children were asked to read the two numbers from left to right and judge whether the number pair was in the “correct” (i.e., ascending from left to right) or “incorrect” counting order. In one block, children were asked to press “F” with their left index finger if the numbers were in the correct order and “J” with their right index finger if the numbers were not. In the other block, the assignment of response keys was reversed with the “J” key representing a correct order and the “F” key representing an incorrect order. Before the formal experiment, there were eight practice trials. The proportion of problems solved correctly indexed children’s performance.

##### Number sense task

The non-symbolic magnitude comparison adapted from Ginsburg and Baroody (1990) was used to assess children’s number sense. Children were asked to estimate (without counting) which of the two sets of dots, presented simultaneously on the screen, contained more dots (36 trials, 5 s per trial). The number of dots varied from 5 to 12, and the ratios were 2:3, 5:7, and 3:4. Dots differed in size, but the total combined area of all dots in each set was controlled to be the same. Children were required to press the “Q” key with their left index finger when there were more dots on the left or press the “P” key with their right index finger when there were more dots on the right. The proportion of problems solved correctly indexed children’s performance.

##### Number line estimation task

This task was adapted from Booth and Siegler (2006). Children were instructed to locate 26 numbers in the number axis (range: 0–100). Each number was presented only once. In each trial, a horizontal line appeared on the screen with the left endpoint labeled “0” and the right endpoint labeled “100.” Each child was required to either mark the presented number position on the 0–100 axis with the mouse or point to the location with his/her finger (some children could not use the mouse). This task had no time limit. Each number appeared on the left side above the line. The 26 numbers presented were 3, 4, 6, 8, 12, 14, 17, 18, 21, 24, 25, 29, 33, 39, 42, 48, 52, 57, 61, 64, 72, 79, 81, 84, 90, and 96. Their order was randomized for each child. The computer accurately recorded the children’s responses. The score on this test was calculated in terms of accuracy using the following formula (Cui et al., 2017): Accuracy = 100 – (response – standard answer)/(standard answer + [response – standard answer]) × 100. The formula returns values from 0 to 100. *Response* refers to the child’s answer, and *standard answer* refers to the correct answer. Deviation of a child’s answer from the standard answer is divided by the sum of the standard answer and the deviation, which gives the degree of deviation from the standard value. The formula was adapted from the formula for the percentage absolute error (PAE) (Siegler and Mu, 2008): PAE = (estimate – estimated quantity)/scale of estimates. Given that the children could provide any number as the solution in some cases, there was no limit on their responses. To address this issue, the denominator in Siegler and Mu’s formula was revised. The final score for each child was the average accuracy of all trials.

#### Statistical Analysis

To examine whether children with high finger gnosis differed from their peers with low finger gnosis in basic number processing abilities, a series of ANOVAs were conducted, taking the group as a between-subjects factor and four basic number processing abilities as dependent variables. In addition, the age was a covariant in all ANOVAs. Finally, path analysis was carried out to test the potential mediation effect of basic number processing abilities in the relationship between finger gnosis and addition skills. Data analysis was executed in SPSS 21.0.

### Results

The ANOVA results revealed a significant difference between children with high finger gnosis and their peers with low finger gnosis in the number line estimation task even after Bonferroni–Holm correction, *F*(1, 33) = 4.003, *p* = 0.054, Cohen’s *d* = 0.980. Children with high finger gnosis performed better on the number line task than their peers with low finger gnosis. Conversely, no significant difference was found between the two groups in enumeration, *F*(1, 32) = 0.16, *p* = 0.693; order judgment, *F*(1, 33) = 0.23, *p* = 0.632; and number sense, *F*(1, 33) = 0.12, *p* = 0.731. Descriptive statistics appear in Table 4.

**TABLE 4:** Descriptive statistics in Study 2.

	Children with low finger gnosis		Children with high finger gnosis	
		
	*M*	*SD*	*M*	*SD*
Enumeration task	81.67	11.12	85.05	12.78
Order judgment task	86.67	8.99	89.20	7.05
Number sense task	80.90	11.38	84.31	12.66
Number line estimation task	71.38	9.97	79.80	7.90
Finger gnosis	62.25	7.39	91.05	3.49
Addition accuracy	69.13	28.00	81.65	18.10

**Legend for all tasks is the percentage of correct trials (%); percentage for number line estimation task is based on the following formula (Cui et al., 2017): Accuracy = 100 – (response – standard answer)/(standard answer + [response – standard answer]) × 100.**

Based on the discussed results, a path model was estimated to test whether the correlation between finger gnosis (X) and addition accuracy (Y) was mediated by number line estimation (M). Mediation was assessed using the process outlined in Preacher and Hayes (2008). Partial correlation results with age as a covariant are presented in Table 5.

**TABLE 5:** Correlations among finger gnosis, addition skills, and number line estimation after controlling for age.

	*M*	*SD*	Addition accuracy	Finger gnosis	Number line estimation
Addition accuracy	76.08	23.53	−		
Finger gnosis	78.25	15.51	0.238	–	
Number line estimation	76.06	9.72	0.435**	0.408*	–

*p < 0.05. **p < 0.01.

**FIGURE 1:**
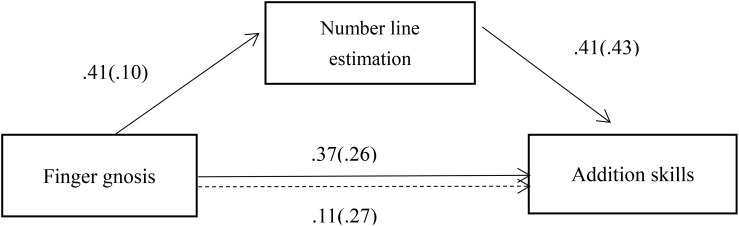
Solid line indicates a significant direct path from finger gnosis to number line estimation, from finger gnosis to addition skills, and from number line estimation to addition skills. Dotted line denotes a non-significant path from finger gnosis to addition skills when controlling for number line estimation. Standardized OLS regression coefficients and standard errors (in brackets) were on the path.

The first step estimated the effect of finger gnosis on addition accuracy (i.e., c-path or X→Y relationship). The second step estimated the effect of finger gnosis on number line estimation accuracy (i.e., a-path or X→M relationship). The third step estimated the effect of number line estimation on addition accuracy (i.e., b-path or M→ Y relationship), controlling for the independent variable (X). The effect of X on Y in the third step constituted the c’-path (i.e., change in the outcome not explained by the mediator). Finally, the indirect effect was calculated as the product of a and b estimates, denoted as ab. When ab is significant, then the mediation path proposed in the research hypothesis exists (Preacher and Hayes, 2008). A bias-corrected bootstrap-confidence interval (CI) for the product of these paths that does not include zero suggests a significant indirect effect (Preacher and Hayes, 2008). As seen in Figure 1, using the INDIRECT procedure with 5,000 bootstrap samples taking age as a covariate revealed a significant positive indirect effect of finger gnosis on addition accuracy through number line estimation (effect = 0.258, 95% CI = 0.0018–0.5835). Moreover, when controlling for the mediating variable, the direct effect of finger gnosis on addition accuracy was not significant (*B* = 0.113, *p* = 0.680, 95% CI = −0.4383 to 0.6635). This finding suggests that number line estimation played a fully mediating role in the relationship between finger gnosis and addition accuracy.

### Discussion

Study 2 indicated that children with high finger gnosis performed better in number line estimation than their counterparts with low finger gnosis. More importantly, number line estimation fully mediated the correlation between finger gnosis and addition skills. These findings imply that number line estimation may underlie the relationship between finger gnosis and addition skills. In other words, children with higher finger gnosis may develop a more mature mental number line, which helps them perform better on addition tasks. Study 2 also showed that children with high finger gnosis did not differ from their peers with low finger gnosis in enumeration, order judgment, or number sense. This result suggests that these number processing abilities cannot explain why finger gnosis is relevant to addition problem-solving.

## General Discussion

This study examined for the first time whether finger gnosis was associated with addition skills in young Chinese children. Results revealed two noteworthy findings. First, finger gnosis was associated with addition performance in 5–6-years-old Chinese children, and this correlation persisted after controlling for children’s age. Second, the relationship between finger gnosis and addition performance was fully mediated by number line estimation. Moreover, we found that girls performed better in finger gnosis than boys, and children who had musical training performed better than their peers who had no musical training. We discuss the underlying reasons for these findings and their important implications later.

In line with most previous research (e.g., Fayol et al., 1998; Noël, 2005; Gracia-Bafalluy and Noël, 2008; Penner-Wilger and Anderson, 2008; Reeve and Humberstone, 2011), our results suggest that finger gnosis explains a unique and substantial proportion of variance in young children’s addition skills.

One important finding from the present research is that the association between finger gnosis and addition skills seems to be fully mediated by number line estimation. Number line estimation is closely associated with mathematical competence (see a meta-analysis by Schneider et al., 2018). Typically, number line estimation is regarded as an indicator of numerical representations (e.g., Siegler and Booth, 2004; Opfer and Siegler, 2007; Booth and Siegler, 2008; Siegler and Ramani, 2008). Recently, performance on the number line task is highly correlated with visuospatial skills (Gunderson et al., 2012; Lefevre et al., 2013; Thompson et al., 2013; Crollen and Noël, 2015a). Furthermore, the relationship between number line estimation and mathematical achievement can be fully explained by visuomotor integration and visuospatial skills (Simms et al., 2016). Numerical representations or visuospatial skills that underlie number line estimation likely drive the correlation between finger gnosis and addition skills. Fingers are highly important for several related tasks, including understanding the cardinal meaning of number words (Butterworth, 1999), establishing the one-to-one correspondence principle (Gallistel and Gelman, 1992), and mapping the symbolic system onto the preexisting non-symbolic, spatial magnitude system (Fayol and Seron, 2005). Therefore, strong finger gnosis may help children to acquire number knowledge and to establish number and spatial representations (Noël, 2005; Penner-Wilger and Anderson, 2013), which are essential to the development of arithmetic skills.

Finger gnosis may also facilitate children’s development of spatial skills. Recently, Soylu et al. (2018) contended that the finger gnosis task measures one’s ability to activate an internal body representation and then map that spatial representation onto external objects. In other words, the spatial representation underlying finger gnosis can influence arithmetic skills. Indeed, Newman (2016) identified a strong correlation between finger sense and matrix reasoning, which involves a series of figures representing a pattern with one figure left blank. Potentially, children with higher finger gnosis tend to develop stronger spatial skills, which facilitates a more mature mental number line and better arithmetic skills.

Our findings can be reconciled with weak associations between finger gnosis and arithmetic skills from studies by Poltz et al. (2015) and Wasner et al. (2016). In Wasner et al. (2016), general cognitive ability was measured using continuing rows and matrices subtests from the Culture Fair Intelligence Test—Revised (Weiß and Osterland, 2013). The two subtests measure children’s visual–spatial reasoning abilities. Therefore, in Wasner et al. (2016), the correlation between finger gnosis and arithmetic was likely overridden by general cognitive ability. Similarly, in Poltz et al. (2015), the relationship between finger gnosis and calculation was likely overridden by non-verbal intelligence, which, in fact, measures children’s visual–spatial abilities. As for the study by Long et al. (2016), the association between finger gnosis and arithmetic skills may have been overridden by non-symbolic magnitude judgment skills, which may also involve visuospatial abilities as suggested in recent studies (Burr and Ross, 2008; He et al., 2015).

Our finding that the association between finger gnosis and addition skills was fully mediated by number line estimation may provide some support for the redeployment hypothesis. Finger gnosis circuit may share some circuits with number line estimation, which is redeployed to support complex arithmetic skills. In other words, the functional overlaps between finger gnosis and number line estimation provide strong support for addition skills. Specifically, fingers have an ordinal meaning that is determined by a finger’s specific position within the counting sequence (Sixtus et al., 2020). Finger gnosis is typically shaped by counting a number on 10 fingers, which involves the successor and predecessor knowledge in the number sequence (Sella and Lucangeli, 2020). In this sense, finger gnosis can scaffold number line estimation, which involves placing a number on the number sequence. Both finger counting and number line are powerful conceptual structures to unfold the understanding of the magnitude relation between symbols and constitute the basis for building the first arithmetical operations (Sella et al., 2020). However, children seem to rely more on a spatial organization than on counting to achieve a full understanding of the magnitude relations between digits (Sella et al., 2017). Number line estimation has been suggested to involve the ability to accurately divide space and/or numbers (e.g., Berteletti et al., 2010; Barth and Paladino, 2011; Ashcraft and Moore, 2012; Rouder and Geary, 2014) as well as one’s ability to judge the scale of a line and to parse the space into segments (Simms et al., 2016). These abilities are similar to arithmetic addition and subtraction, which involve adding parts to make a whole or dividing a whole into parts. Therefore, the circuits for number line estimation could potentially be redeployed for addition skills.

In the present research, finger gnosis was not correlated with children’s use of a finger-counting strategy when solving addition problems. Finger gnosis may represent a domain-general ability that develops in finger-use activities, including counting as well as handcrafting. By contrast, finger counting is a domain-specific strategy used only in arithmetic problem-solving. Therefore, when facing an addition task, children with good finger gnosis do not necessarily use a finger-counting strategy. According to the two developmental stages proposed by Reeve and Humberstone (2011), many children in our study may have been in the second stage in which they could flexibly and adaptively use their fingers; that is because they could use other more economic strategies such as memory retrieval; they did not resort to finger counting. Chinese families also tend to emphasize children’s rote memorization of arithmetic facts. When facing addition and subtraction problems, children are encouraged to provide answers as quickly as possible, which may lead children to shift from relying on finger counting to memory retrieval. In addition, many kindergartens in China teach primary-school-level lessons, including addition and subtraction. It is thus unsurprising that 25.8% of children in this study used retrieval strategies, which can predict their addition performance. During children’s addition skill development, retrieval gradually becomes a dominant strategy compared with finger counting. In turn, as revealed by some previous studies, the use of finger-counting strategies may be negatively associated with later mathematics achievement (Fennema et al., 1998; Geary et al., 2004; Carr and Alexeev, 2011).

In the present research, children’s sex and experience playing musical instruments explained some individual differences in finger gnosis: girls were better in finger gnosis than boys. This sex difference might be due to the distinct games boys and girls play in early childhood. Girls generally prefer games that involve their fingers (e.g., handicrafts and dressing up dolls). By contrast, boys prefer games that require little fine finger participation (e.g., basketball and toy guns). In this sense, finger training among girls might be greater than among boys. It is, therefore, not surprising that girls had better finger gnosis than boys. In addition, our research indicated that children who had played finger instruments, including piano, guitar, and flute, had better finger gnosis than those who had not. This finding suggests that playing musical instruments may be somewhat helpful for improving finger gnosis. In other words, playing musical instruments may indirectly and positively influence children’s addition skills. Previous studies have found that musical training can promote mathematical abilities such as number conception, addition, and subtraction to a certain extent (e.g., Cheek and Smith, 1999; Cabanac et al., 2013). Finally, our study revealed that boys were more likely to use retrieval in solving addition tasks than girls. This finding is consistent with prior work (e.g., Carr and Jessup, 1997; Fennema et al., 1998; Carr and Davis, 2001). One explanation is that boys are more influenced by perceived adult beliefs or actions than girls (Carr et al., 1999). Because boys believe that adult-like strategies are reflective of ability, they may be more heavily influenced by teacher instructions regarding retrieval strategies. Conversely, girls’ strategy use may be less affected by adults.

It should be noted that the finding that girls with high levels of finger gnosis did not have better addition performance than boys can be reconciled with the conclusion of a positive relationship between finger gnosis and addition performance. Indeed, girls had a higher level of finger gnosis than boys. However, boys used retrieval strategies more frequently than girls. Both the frequency of retrieval strategy and finger gnosis predicted arithmetic performance (see Table 3). Therefore, high levels of finger gnosis might have counterbalanced infrequent use of retrieval strategy for girls. As a result, they did not show better addition performance than boys. Similarly, frequent use of retrieval strategy might have counterbalanced low levels of finger gnosis for boys, which may explain why boys did not show better addition performance than girls.

Certain limitations of this study should be noted. First, in Study 2, we selected only children with high and low finger gnosis due to practical limits and ignored those with moderate finger gnosis. This selection may have reduced the statistical power of Study 2. Future studies should use a representative sample to replicate our research findings. Second, the present research is cross-sectional; longitudinal and training studies are necessary to establish prospective and causal relations between finger gnosis and children’s arithmetic skills.

## Conclusion

In conclusion, the findings of this study enhance our understanding of the correlation between finger gnosis and arithmetic skills. One practical suggestion is that encouraging young children’s finger use may be beneficial, particularly as finger use could be helpful for finger gnosis and thus for children’s numerical and arithmetic development. Therefore, we encourage educators (including teachers and parents) to offer appropriate finger training for their children in their educational practices.

## Data Availability Statement

The datasets presented in this study can be found in online repositories. The names of the repository/repositories and accession number(s) can be found in the article/ Supplementary Material.

## Ethics Statement

The studies involving human participants were reviewed and approved by the Administration Committee of Psychological Research in Southwest University and in compliance with the ethical guidelines of the American Psychological Association. Written informed consent to participate in this study was provided by the participants’ legal guardian/next of kin.

## Author Contributions

LZ conceived and designed the experiments. WW performed the experiments and analyzed the data. LZ and XZ wrote the manuscript. All authors contributed to the article and approved the submitted version.

## Conflict of Interest

The authors declare that the research was conducted in the absence of any commercial or financial relationships that could be construed as a potential conflict of interest.

## Footnotes

1 www.dweipsy.com/lattice
